# Diagnosis of Atelosteogenesis Type I suggested by Fetal Ultrasonography and Atypical Paternal Phenotype with Mosaicism

**DOI:** 10.1055/s-0038-1670684

**Published:** 2018-09

**Authors:** Joanna Goes Castro Meira, Manoel Alfredo Curvelo Sarno, Ágatha Cristhina Oliveira Faria, Guilherme Lopes Yamamoto, Débora Romeo Bertola, Gabriela Gayer Scheibler, Dione Fernandes Tavares, Angelina Xavier Acosta

**Affiliations:** 1 Department of Medical Genetics, Hospital Universitário Professor Edgard Santos, Universidade Federal da Bahia, Salvador, BA, Brazil; 2 Maternidade Climério de Oliveira, Universidade Federal da Bahia, Salvador, BA, Brazil; 3 Center for Studies of the Human Genome and of Stem Cells Department of Genetics and Evolutionary Biology, Institute of Biosciences, Universidade de São Paulo, São Paulo, SP, Brazil

**Keywords:** atelosteogenesis, somatic mosaicism, skeletal dysplasia, *FLNB*, exome-target sequencing, fetal ultrasonography, atelosteogênese, mosaicismo somático, displasia esquelética, *FLNB*, sequenciamento do exoma, ultrassonografia fetal

## Abstract

Atelosteogenesis type I (AOI) is an autosomal dominant skeletal dysplasia caused by mutations in the *filamin* *B* (*FLNB*) gene with classic and well-recognizable clinical findings. However, parents affected with a mild phenotype, probably with somatic mosaicism, can generate offspring with a much more severe phenotype of AOI. In the present report, we describe a female newborn with classic AOI leading to early neonatal death, whose diagnostic was based on prenatal radiological findings and on the physical examination of the father. Since her father had limb deformities and corporal asymmetry, suggesting somatic mosaicism, his biological samples were analyzed through a gene panel for skeletal dysplasias. A missense mutation not previously described in the literature was detected in the *FLNB* gene, affecting ∼ 20% of the evaluated cells and, therefore, confirming the diagnosis of mosaic AOI in the father. The molecular analysis of the father was crucial to suggest the diagnosis of AOI in the newborn, since she died early and there were no biological samples available.

## Resumo

A atelosteogênese tipo I (AOI) é uma displasia esquelética autossômica dominante causada por mutações no gene *filamina B* (*FLNB*) com achados clínicos clássicos e bem reconhecíveis. No entanto, pais afetados com um fenótipo mais leve, provavelmente com mosaicismo somático, podem gerar uma prole com um fenótipo muito mais grave de AOI. No presente relato, descrevemos um recém-nascido do sexo feminino com AOI clássica, que levou à morte neonatal precoce, e cujo diagnóstico foi baseado em achados radiológicos pré-natais e no exame físico de seu genitor. Como o genitor apresentava deformidades em membros e assimetria corporal, que sugeriam mosaicismo somático, suas amostras biológicas foram analisadas por meio de um painel de genes para displasias esqueléticas. Uma mutação *missense*, não descrita anteriormente na literatura, foi detectada no gene *FLNB*, afetando ∼ 20% das células avaliadas, e, portanto, confirmando o diagnóstico de AOI em mosaico no genitor. A análise molecular realizada no genitor foi fundamental para sugerir o diagnóstico de AOI na recém-nascida, uma vez que esta morreu precocemente, e não havia amostras biológicas disponíveis.

## Introduction

Atelosteogenesis type I (AOI) is a disease of autosomal dominant inheritance associated with mutations in the *filamin B* (*FLNB*) gene, located on chromosome 3p14, which encodes the filamin B protein.^1^ This syndrome includes a spectrum of phenotypes that may vary from mild, such as Larsen syndrome (LS) and spondylocarpotarsalsynostosis (STC), to severe conditions, such as atelosteogenesis type III (AOIII), and boomerang dysplasia. Atelosteogenesis type I, also known as giant cell chondrodysplasia or spondylohumerofemoral hypoplasia, presents as its main manifestation the disordered and incomplete ossification of the skeleton, and it is characterized by severely shortened limbs, displaced hips, knees and elbows, and club feet.^2^ Its radiographic features include pelvic hypoplasia; absent, reduced or distally sharpened humerus and femurs; shortened or curved ulna and tibia; absent fibulae; metacarpals and middle and proximal phalanges without ossification or partially ossified; and high perinatal lethality.^3^

Some studies suggest that AOI and AOIII, a chondrodysplasia first described in 1991 with clinical and radiological characteristics similar to AOI, appear to represent a continuous phenotype.^3^ ^4^ Unlike AOI, which is highly lethal, AOIII is clinically milder, and usually the affected individual survives after the neonatal period. The clinical picture of AOIII is recognizable from birth, and is characterized by rhizomelic shortening, joint dislocations, club feet, broad nails, polysyndactyly, narrow chest, ocular hypertelorism, flat nasal bridge, micrognathia, and cleft palate. Its radiographic features include distal tapering of the humerus and femurs, short and broad tubular bones in the hands and feet, and mild vertebral hypoplasia. Besides that, the affected children may present respiratory failure due to laryngotracheomalacia and thoracic hypoplasia, indicating that cases of children with AOIII, whose parents had milder phenotypes (similar to Larsen syndrome), occur probably as a result of parental mosaicism, while the children, due to a germline mutation, have all of their cells affected by the mutation and, therefore, present a much more severe phenotype of the disease.^5^ ^6^ In the present report, we describe the process of diagnosis of AOI and the variability of the clinical findings in two patients, a father and his child.

## Case Report

**Fig. 1:**
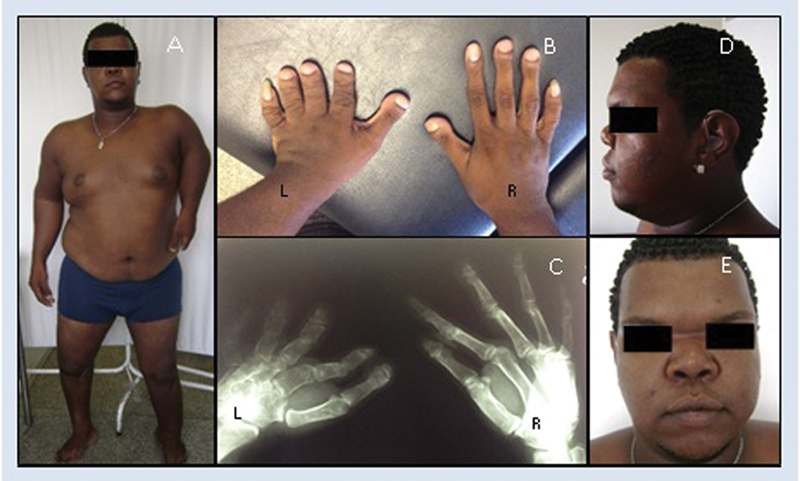
(**A**): The father showing disproportionate short stature with short and asymmetrical legs, shortened upper left limb with restricted elbow extension; genum varum on the right. (**B**, **C**) Asymmetry of the hands, left hand with brachydactyly, wide distal phalanges, spatulate fingers. (**D**, **E**) Macrocephaly, ocular hypertelorism, low nasal root, short nasal dorsum, anteverted nostrils, prominent supraorbital ridges.Note: images provided and authorized by parents with informed consent.

A 13-year-old pregnant woman was referred to a geneticist after an abnormal second-trimester gestational ultrasonography showing fetal dysmorphisms. The gestational ultrasonography showed a fetus presenting marked shortening of the femurs, humeral agenesis, micrognathia, nasal hypoplasia, ocular hypertelorism, thoracic scoliosis, and hypoplastic ribs. The father of the fetus was a 29-year-old man with limb deformities and facial dysmorphism who had never been subjected to an evaluation with a geneticist. There were no parental consanguinity or reports of similar cases in the family, and the condition in the father was apparently *de novo*. The father presented disproportionate short stature (with the lower limbs disproportionally short in relation to the trunk), thoracic and scapular asymmetry, scoliosis, macrocephaly, ocular hypertelorism, low nasal root, short nasal dorsum, bifid uvula, prominent supraorbital ridges, shortening of all segments of the left upper limb with brachydactyly and widening of the distal phalanges of this limb, restriction of elbow extension, and lower limb asymmetry and shortening with genu varum (Fig. 1). He also had bilateral conductive hearing loss, and had been submitted to a surgery in the lower limbs, because he was born with the “legs bent.” At this point, it was suggested that the deformities of the newborn girl were inherited from her father.

**Fig. 2:**
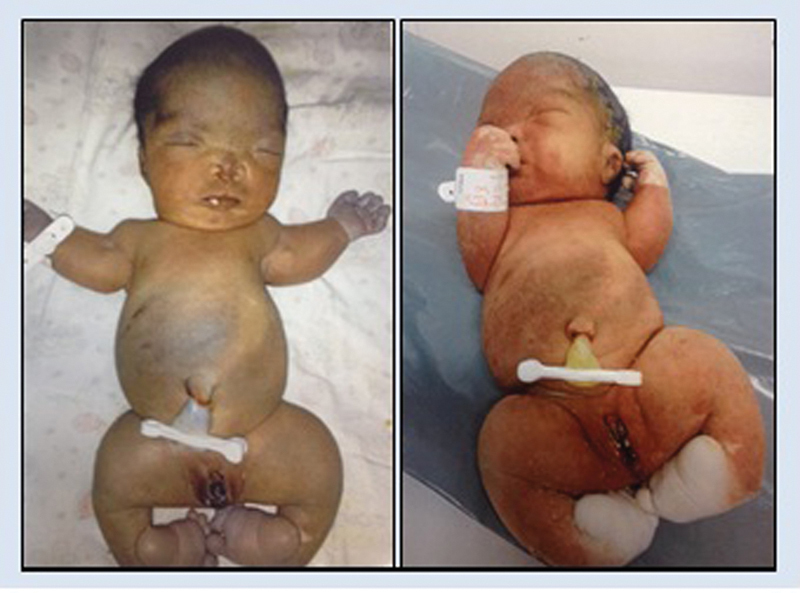
Stillborn with short stature, short and curved femurs, club feet, macrocephaly, broad forehead, hypertelorism, nasal hypoplasia, micrognathia, short neck, humerus agenesis, thoracic narrowing, short and spatulate fingers.Note: images privided and authorized by parents with informed consent.

The newborn died shortly after birth and had multiple skeletal anomalies: marked shortening of the femurs, humeral agenesis, micrognathia, nasal hypoplasia, ocular hypertelorism, thoracic scoliosis, and hypoplastic ribs, in addition to short neck, thoracic narrowing, small hands with shortened and spatulate fingers, legs in a “lotus flower” position with large hallux, and club feet. Unfortunately, the child was born in a maternity hospital in which the medical team did not perform complementary neonatal tests, such as X-rays, and we only had access to the photographs of the newborn (Fig. 2). Due to the impossibility of postmortem exams and the unavailability of biological samples of the newborn, we have opted to carry out the analysis of next generation sequencing (NGS) of a panel of genes associated to the skeletal dysplasias in the affected father.

## Methods

**Samples:** The DNA sample was extracted from the saliva of the father, which was collected with the Oragene DNA Collection Kits OG-500 and OG-575, and purified following prepIT-L2P manufacturer's instructions (DNA Genotek, Ottawa, ON, Canada). For the controls, we used our in-house whole exome sequencing data from 609 Brazilians (Online Archive of Brazilian Mutations, ABraOM: http://abraom.ib.usp.br/), as well as public databases (1000 Genomes Project - http://www.internationalgenome.org; Exome Variant Server/NHLBI ESP exomes - http://evs.gs.washington.edu/EVS/; The Genome Aggregation Database (gnomAD) - gnomad.broadinstitute.org/; and Exome Aggregation Consortium (ExAC) http://exac.broadinstitute.org/).

**Next-generation sequencing target:** An NGS target of a panel of genes associated with skeletal dysplasia (Table 1) was performed with the Illumina MiSeq sequencer (Illumina, San Diego, CA, US), using Illumina's Nextera kits for library preparation. The KAPA Library Quantification kit (KAPA Biosystems, Wilmington, MA, US) was used to quantify the libraries by real-time quantitative polymerase chain reaction (PCR). The sequence alignment, as well as the data processing, variant calling, and variant annotation were performed with Burrows-Wheeler Aligner (BWA) (http://bio-bwa.sourceforge.net), Picard (http://broadinstitute.github.io/picard/), Genome Analysis Toolkit package (GATK) (https://www.broadinstitute.org/gatk/), and ANNOVAR (http://www.openbioinformatics.org/annovar/) respectively.

**Table 1:** List of genes in skeletal dysplasia panel

Genes								
***ACP5***	***COL1A2***	***EIF2AK3***	***FLNB***	***LEMD3***	***OFD1***	***SALL1***	***TCOF1***	***EIF4A3***
***ADAMTS18***	***COL2A1***	***ELN***	***GALNS***	***LEPRE1***	***PAPSS2***	***SATB2***	***TFAP2A***	***PRKAR1A***
***ADAMTSL2***	***COL3A1***	***ERF***	***GDF5***	***LFNG***	***PLCB4***	***SBDS***	***TGFBR1***	***BMP1***
***ALPL***	***COL5A1***	***EVC***	***GDF6***	***LIFR***	***PLOD1***	***SERPINH1***	***TGFBR2***	***CREB3L1***
***ALX1***	***COL5A2***	***EVC2***	***GJA1***	***LMNA***	***POLR1C***	***SH3BP2***	***TGIF1***	***HUWE1***
***ALX3***	***COL9A1***	***EXT1***	***GLB1***	***LRP5***	***POLR1D***	***SHH***	***TNFRSF11A***	***IFITM5***
***ALX4***	***COL9A2***	***FBLN5***	***GLI2***	***MATN3***	***POR***	***SHOX***	***TP63***	***PLOD2***
***ANO5***	***COL9A3***	***FBN1***	***GLI3***	***MESP2***	***PPIB***	***SIX3***	***TRAPPC2***	***PLS3***
***CANT1***	***COMP***	***FGF8***	***GNAI3***	***MMP13***	***PTCH1***	***SLC26A2***	***TRIP11***	***SERPINF1***
***CHST14***	***CRTAP***	***FGFR1***	***GRHL3***	***MMP9***	***PTH1R***	***SMARCAL1***	***TRPS1***	***SPARC***
***CHST3***	***CTSK***	***FGFR2***	***GNAS***	***MSX1***	***PVRL1***	***SOST***	***TRPV4***	***TBX6***
***COL10A1***	***DDR2***	***FGFR3***	***HES7***	***MSX2***	***RAB23***	***SOX9***	***TSHZ1***	***TMEM38B***
***COL11A1***	***DLL3***	***FIG4***	***IFT80***	***NF2***	***RECQL4***	***SP7***	***TWIST1***	***WNT1***
***COL11A2***	***DYM***	***FKBP10***	***IL11RA***	***NKX3–2***	***RMRP***	***TBX1***	***WNT3***	***ZIC1***
***COL18A1***	***DYNC2H1***	***FKBP14***	***IRF6***	***NOG***	***ROR2***	***TBX22***	***ZIC2***	
***COL1A1***	***EFNB1***	***FLNA***	***KIF22***	***NPR2***	***RUNX2***	***TCF12***	***EDN1***	

In filtering, we have considered only rare mutations with a minor allele frequency (MAF;< 0.5%) in all populations of the public databases analyzed and in our in-house control database. Only variants with > 20 read depths, average quality score > 30, allelic balance > 0.90 for the alternative allele and < 0.10 for the reference allele for variants in homozygous, and allelic balance between 0.2 and 0.8 for variants in heterozygous, and with strand bias < 2 were considered. All loss-of-function variants (LoFs; (mutations in splicing sites, frameshifts and stop-gains) were considered pathogenic. Missense variants were considered pathogenic only if predicted to be “possibly damaging” or “probably damaging” by the Polymorphism Phenotyping v2 (PolyPhen-2, Sunyaev lab, Harvard Medical School, Boston, MA, US) software/web server, “deleterious” by the Sorting Intolerant From Tolerant (SIFT) (http://sift.jcvi.org) algorithm, and with the Combined Annotation Dependent Depletion (CADD) (https://cadd.gs.washington.edu/) score reported to be > 15. Candidate variants were manually checked on the Integrative Genomics Viewer (IGV) (Broad Institute, Cambridge, MA, US). Synonymous and untranslated region (UTR) mutations were excluded due to the uncertainty of their functional relevance. The genomic position of the mutations is based on the hg19/GRCH37 version of the human reference genome (Genome Reference Consortium – http://www.ncbi.nlm.nih.gov/projects/genome/assembly/grc/). All of the remaining variants were checked using the American College of Medical Genetics and Genomics (ACMG) guidelines.

## Results

The sequencing analysis of the NGS-target in the father has led to the identification of 5 rare mutations that were predicted to be damaging (Table 2). One of the mutations was a splice site-disrupting single nucleotide, and the remaining were four missenses variants in heterozygosis. The splice site mutation was in the *BRCA2* (c.8488–1G > A) gene, and it was present in only one control of our Brazilian database (ABraOM). It was described by the Single Nucleotide Polymorphism Database (dbSNP) and by the ClinVar database, and was classified by the ACMG guidelines as pathogenic and associated with breast and ovarian cancers. Despite its probable pathogenicity, it is not related with our proband phenotype or not relevant to AOI. Among the missense mutations, only the missense mutation found in heterozygosity in exon 3 of the *FLNB* gene (c.596G > C; p.Arg199Pro) appeared to be the causative mutation of the phenotype of the patient. This variant was not described in any public database; it was predicted as pathogenic and classified as likely pathogenic by the ACMG. As it was present in only 20% of the reads sequenced in this region, we consider that this mutation is presented as mosaic in the patient. The remaining 3 missenses mutations (*LEPRE1*: exon10:c.1477G > C:p.Ala493Pro; *LAMA2*: exon23:c.3379T > C:p.Cys1127Arg; *CANT1*: exon4:c.896C > T:p.Pro299Leu) were not considered causal due to a lack of relevance of the role of the gene for AOI, considering the phenotype of the patient. Furthermore, these mutations were not considered pathogenic or likely pathogenic by the ACMG guidelines, and they are present in our controls, and are related to recessive diseases. The missense variant in the *LEPRE1* gene was classified as likely benign by the ACMG guidelines, and it was present in public databases and in our Brazilian controls. The *CANT1* gene variant is present in gnomAD controls, and was classified as of uncertain significance by the ACMG guidelines. The *LAMA2* gene is associated with muscular dystrophy, although it is not described in any public database.

**Table 2:** List of mutations found after performing the exoma sequencing of the affected father

Chr	Gene	Type of variant	Variant	ACMG guidelines	dbSNP	PolyPhen-2	SIFT
chr1	*LEPRE1*	nonsynonymous SNV	NM_001146289:exon10:c.G1477C:p.A493P	Likely benign	rs201977455	Probably damaging	deleterious
chr3	*FLNB*	nonsynonymous SNV	NM_001164317:exon3:c.G596C:p.R199P	Likely pathogenic	NA	Possibly damaging	deleterious
chr6	*LAMA2*	nonsynonymous SNV	NM_000426:exon23:c.T3379C:p.C1127R	Uncertain significance	NA	Probably damaging	deleterious
chr13	*BRCA2*	splicing	NM_000059:exon20:c.8488–1G > A	Pathogenic	rs397507404	NA	NA
chr17	*CANT1*	nonsynonymous SNV	NM_138793:exon4:c.C896T:p.P299L	Uncertain significance	rs267606700	Probably damaging	deleterious

Abbreviations: ACMG, American College of Medical Genetics and Genomics; Chr, chromosome; dbSNP, The Single Nucleotide Polymorphym Database; PolyPhen-2, Polymorphism Phenotyping v2; NA, not available; SIFT, sorting intolerant from tolerant; SNV, single nucleotide variant.

## Discussion

The prenatal diagnostic hypothesis of AOI is based on radiological imaging tests, such as fetal ultrasonography, which detects most skeletal anomalies around the second trimester of pregnancy. As a lethal skeletal dysplasia, this illness is more amenable to prenatal diagnosis due to an earlier onset with more severe phenotypic features.^7^ The typical findings include limb shortening, thoracic hypoplasia, and underossification of the long bones.^5^ ^8^ During this investigation, we were not able to obtain X-rays or biological samples of the newborn, which would have helped with the variant classification. The clinical investigation of stillbirths presenting dysmorphia or birth defects, including the performance of complementary exams and the collection of biological samples, is still not widespread among health professionals in Brazil. Therefore, the chance to perform a more accurate diagnosis is lost, and, in many cases, the genetic counseling of the parents is not performed. In our report, the fetal ultrasonography and postmortem photographs served as a basis for the AOI hypothesis in the father of the newborn, whose diagnosis was confirmed through molecular analysis of the *FLNB* gene. The missense mutation found in exon 3 of the *FLNB* gene (c.596G > C;p.Arg199Pro), which has not been previously described, is present in 20% of the reads, suggesting a case of somatic mosaicism. This finding confirms the clinical diagnosis of AOI both in the father and in the daughter. The majority of the mutations reported in AOI are in exons 2 to 5 of the *FLNB* gene.^5^ Besides that, mutations in the same protein domain were previously described in cases of AOI and AOIII, and both skeletal dysplasias are characterized by overlapping clinical findings. The lethal presentation of the disease suggests the diagnosis of classic AOI in the newborn, and the same disease, as a somatic mosaicism, in the father. This would mean that not all of the father's cells are affected by the same mutation, therefore explaining the milder and asymmetrical expression and his survival beyond the life expectancy of the disease. Since it is a case of mosaicism, another tissue should be tested, ideally, in order to obtain more information about the level of mosaicism (such as skin and blood), but we did not have access to other samples.

## Conclusion

The clinical features of the father are characteristic of a somatic mosaicism. This becomes evident due to the milder and asymmetrical involvement of his limbs and trunk. However, his gonadal cells were affected by the mutation, which explains the birth of an affected newborn with the complete and lethal phenotype of AOI. In these cases, the transmission pattern is heterogeneous, depending on the proportion of gonadal cells affected in the parent, but it can be considered autosomal dominant. The diagnostic suggestion of AOI in the newborn was only possible through a molecular analysis of selected genes for skeletal dysplasias in her father. It is essential to emphasize the important role of the clinical investigation of a newborn or stillborn with birth defects to establish a syndromic diagnosis. This will allow professionals to perform genetic counseling to the parents. In our case, prenatal and family information were essential to establish the diagnosis because of the lack of neonatal information. The present case report reveals the importance of the prenatal evaluation of the fetus, of the assessment of the family history, and of the role of NGS and selected panels for the etiological confirmation of the disease.
